# Ultrafast electron imaging of surface charge carrier dynamics at low voltage

**DOI:** 10.1063/4.0000007

**Published:** 2020-03-30

**Authors:** Jianfeng Zhao, Osman M. Bakr, Omar F. Mohammed

**Affiliations:** Division of Physical Science and Engineering, King Abdullah University of Science and Technology, Thuwal 23955-6900, Saudi Arabia

## Abstract

The performance of optoelectronic devices strongly depends on charge carrier dynamics on top of surfaces of the absorber layers. Unfortunately, this information cannot be selectively probed using conventional ultrafast laser spectroscopic methods, due to the large penetration depth (tens of nm to *μ*m) of the photon pulses in the pump-probe configurations. Therefore, ultrafast time-resolved approaches that can directly and selectively visualize the behavior of the surface carrier dynamics are urgently needed. Here, we introduce a novel methodology of low-voltage scanning ultrafast electron microscopy that can take ultrafast time-resolved images (snapshots) of the surface of materials at the sub-nanometer level. By this approach, the surface of the photoactive materials is optically excited and imaged, using a pulsed low-voltage electron beam (1 keV) that interacts with the surface to generate secondary electrons with an energy of a few eV, and that are emitted only from the top surface of materials, providing direct information about the carrier dynamics and the localization of electron/holes in real space and time. An outlook on the potential applications of this low voltage approach in different disciplines will also be discussed.

## INTRODUCTION

Surface engineering, such as surface modification,^1^ passivation,^2,3^ and activation,^4^ is one of the most effective strategies for significantly improving interfacial properties and the power conversion efficiency of optoelectronic devices. However, optimizing the processing requires an accurate evaluation of the surface/interface properties of photoactive materials at the atomic and nanoscale levels, which is usually limited or impossible to access using conventional characterization methods, including time-resolved laser spectroscopy.

Several pump-probe methods have been proposed and employed to investigate materials' interfacial properties^5–13^ upon photoexcitation but they are lacking of the superior surface selectivity and high spatiotemporal resolution.^14^ For instance, long distance (∼600 nm) carrier transportation in the perovskite thin film has been directly imaged by ultrafast transient absorption microscopy.^13^ Although this powerful technique with relatively high spatiotemporal resolutions of 50 nm and 200 femtoseconds (fs) has been used to visualize charge carrier dynamics in many materials,^15–17^ it is still used as the conventional ultrafast absorption spectroscopy^18–20^ with its intrinsic characteristic of deep penetration (tens of nm to a few *μ*m),^21^ which provides dynamical information mainly from the bulk, not the surface. Optical-pump-terahertz (or THz)-probe spectroscopy has also been employed to track carrier dynamics and mobility in photovoltaic materials.^22^ For instance, it has been used to track the change in the conductivity of epitaxial graphene and further identify the sequential time scales of ultrafast carrier relaxation-recombination dynamics, benefiting from the interdependency between conductivity and carrier concentration and distribution in graphene. This method provides excellent temporal resolution (∼100 fs) but no spatial resolution.^10^ An optical laser pump-transient photocurrent/photovoltage probe configuration has been developed to map the surface topology indirectly and further investigate the impact of surface defects on the performance of photovoltaic devices as well, but its spatiotemporal resolutions are limited to the *μ*m and *μ*s scales.^9^ These methods offer valuable information about charge carrier dynamics and recombination channels, but the information obtained is either primarily from the bulk rather than the surface or lacks of adequate spatiotemporal resolution.^23^ Therefore, new approaches are urgently needed that enable the characterization of surface morphology and that track surface carrier diffusion/recombination selectively with satisfactory resolution in space and time.

**FIG. 1.**
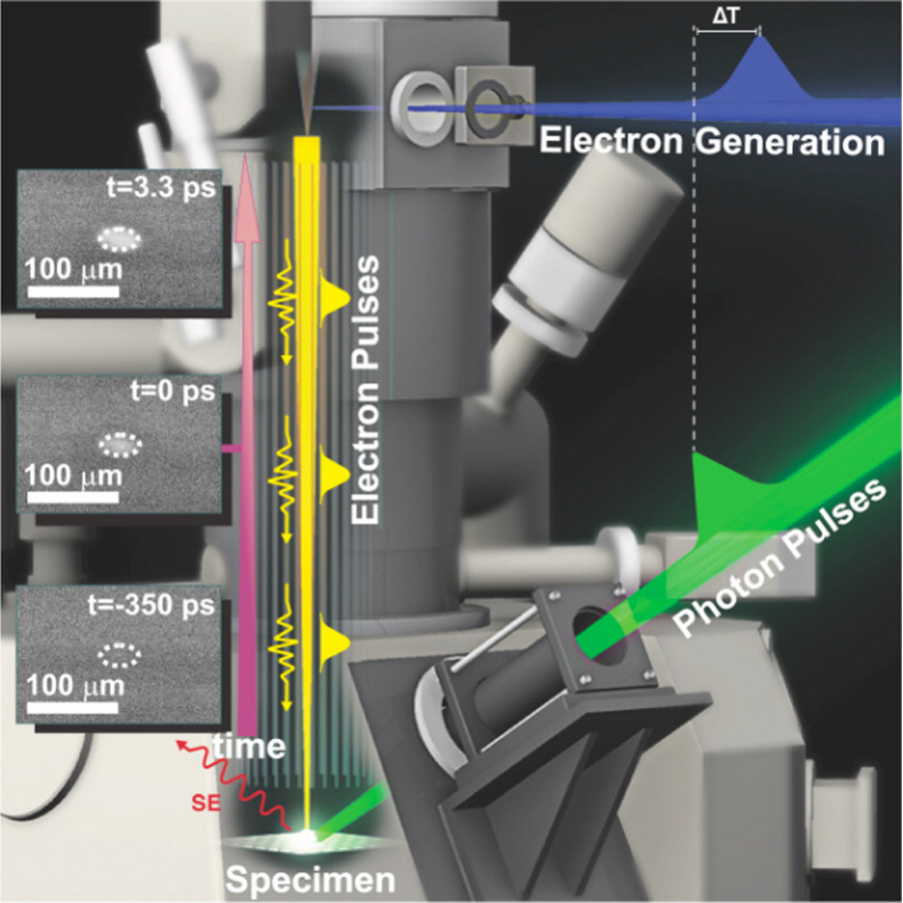
Simplified conceptual schematic of S-UEM. An adjustable optical delay line (not shown) is employed to control the time difference between pump photon pulses and probe primary electron (PE) pulses. Reprinted with permission from Sun *et al.*, J. Phys. Chem. Lett. **6**, 3884 (2015). Copyright 2015 American Chemical Society.^34^ Further inquiries related to the reproduction of Fig. 1 should be directed to the ACS.

Time-resolved electron imaging techniques with microsecond or sub-microsecond temporal resolution in transmission mode (transmission electron microscopy, TEM)^24^ and scanning mode (scanning electron microscope, SEM)^25^ of operation have been developed and used for decades, however, the Zewail group's work at CALTECH on ultrafast electron microscopy (UEM) was a major breakthrough in improving the time resolution down to fs scale,^26–32^ providing a very powerful approach for directly imaging of materials' surface dynamics and structural transitions in real time and space. It is worth mentioning that this state-of-the-art technology initially offered the transmission mode (UEM) in 2005;^26^ the scanning mode (S-UEM) emerged in 2010^29^ and developed rapidly to pursue better spatiotemporal resolution.^33–38^ As shown in Fig. 1, two optical pulses generated by the fundamental output (1030 nm) of a fiber laser system (Clark-MXR) are integrated with modified scanning/transmission electron microscopy in this unique technology. The first optical beam at 515 nm (pump pulse) excites the specimen and initiates the carrier dynamics while the other optical beam (343 nm) is carefully set to strike the field emission gun of an SEM^34^ (Fig. 1) or the photocathode in a TEM,^26^ generating short electron packets through the photoelectric effect. These pulsed electron packets, rather than the thermally generated continuous electron beams in conventional electron imaging, act as the probe. Both modes of UEM retain the fs temporal resolution that originates in the ultrafast laser system and retain their atomic or nm spatial resolutions for conventional transmission and scanning electron microscopy. The main differences between these two UEMs are varying acceleration voltages [AV, typically 1–30 kilovolts (kV) in scanning mode (S-UEM) and 30–300 kV in transmission mode (UEM)] to visualize materials' surface dynamics,^39–45^ surface potentials,^46^ surface mechanical properties^47^ in scanning mode, and structural/phase transitions or intermediate states in transmission mode.^48–53^

Despite the excellent spatial resolution (down to the atomic level) of UEM, its high AV and transmission characters make it incapable of tracking the ultrafast photophysical processes that normally occur near the materials' surfaces/interfaces. S-UEM is demonstrably superior in this regard. For example, the secondary electron (SE) snapshots in Fig. 1 show the generation and diffusion trajectories of photoexcited carriers at the surface of a Si single crystal investigated by S-UEM at 30 kV.^34^ Another advantage of SEM is that the AV, ranging from 1 to 30 kV, can be easily adjusted, providing surface dynamical information at diverse escape depths. In addition, SEM at a low voltage is suitable for characterizing specimens that are sensitive to electron beams or that lacking of high conductivity,^54–56^ which extends the scope of its material characterization applications. In addition to its easier sample preparation [in contrast to the requirement for thin samples (≤100 nm) in TEM measurements], the thicker samples used in SEM enable efficient dissipation of heat generated by laser radiation, minimizing specimen damage during pump-probe experiments. Finally, systematic analyses of the imaging processing, instrumentation, construction developments, and applications of these two modes of UEM have recently been contributed by Kwon *et al.*,^52,57^ Flannigan *et al.*,^58–60^ Mohammed *et al.*,^61,62^ and their colleagues.

## ULTRAFAST ELECTRON IMAGING AT LOW VOLTAGE IN SCANNING MODE

### Theoretical principle and experimental set-up

High AV, such as 30 kV for S-UEM, enables the generation and emergence of SEs that originate at a shallow depth below the material surface (≤5 nm for most semiconductor materials^63^), which is suitable for detecting the surface dynamics of photoactive materials. However, because some surface engineering is restricted to even less depth above the bulk materials—for example, the native oxide layer on a silicon wafer surface is less than 1 nm;^64^ an ultrathin coating (∼1 nm) of aluminum nitride (AlN) is applied to the surface of carbon nanotubes to significantly stabilize it;^65^ and a sub-nm (0.5 nm) layer of cubic Gallium nitride (GaN) passivated GaAs markedly enhances photoluminescence.^66^ These popular surface engineering approaches and their effects cannot be easily investigated by S-UEM at 30 kV because of the considerable disparity between the information depth of SE (∼5 nm) and the thicknesses of engineered surface layers (≤1 nm). To overcome these limitations, we focused on modification of the existing second generation S-UEM apparatus that built in KAUST in 2015 and developed low-voltage scanning ultrafast electron microscopy (LVS-UEM) with a highly surface-sensitive capability^67^ and easier mapping without causing serious radiation-induced damage.^68^ LVS-UEM also paves the way for providing detailed surface morphology information, benefiting from the improved yield ratio between SE and backscattered electrons (BSE).^55^ However, the use of low AV may result in a poorer spatial resolution for LVS-UEM, which is the same case in conventional SEM at low-voltages.^69^

**FIG. 2.**
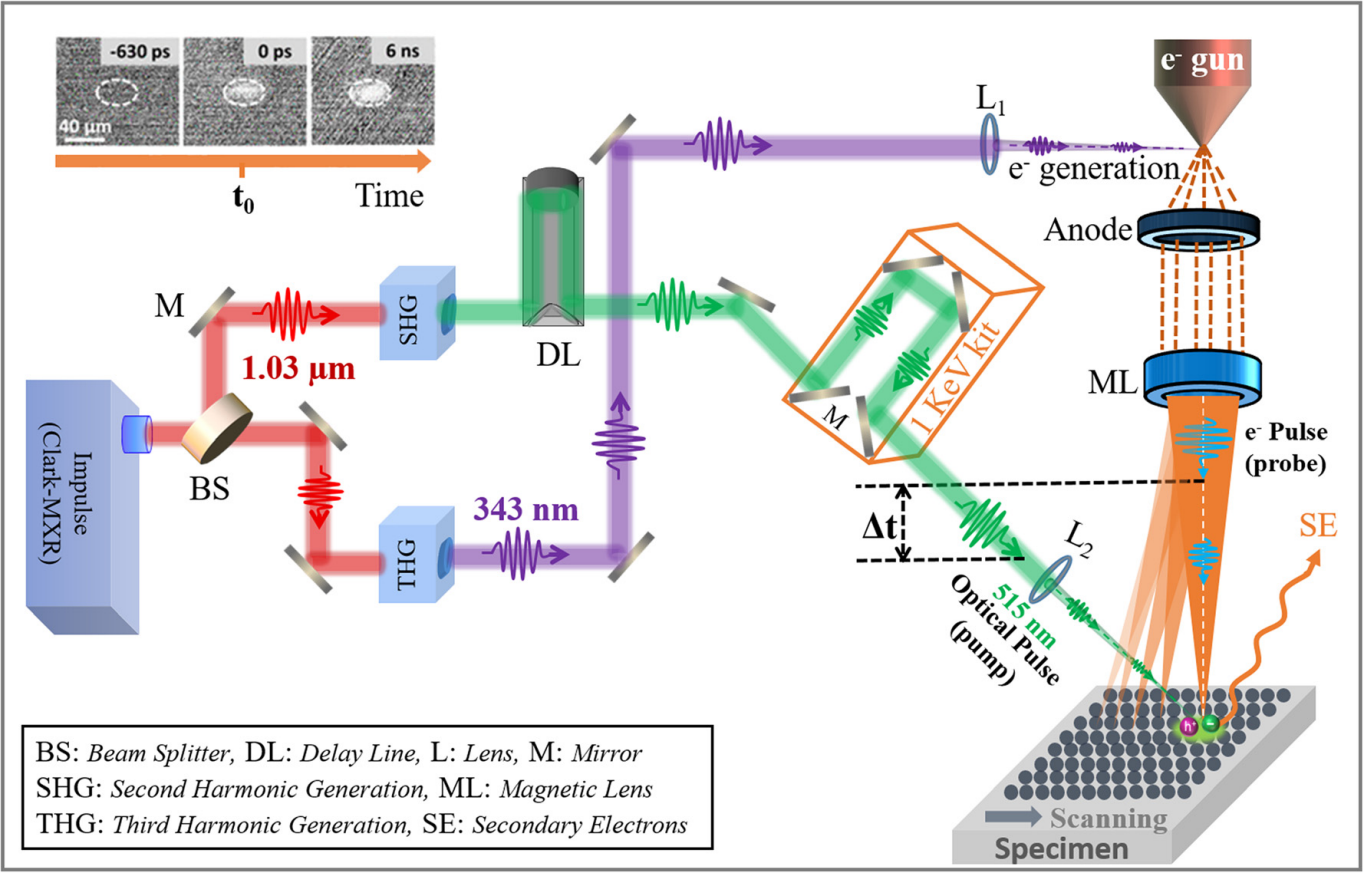
Schematic comparison of S-UEM apparatus at high AV (30 kV) and low AV (1.0 kV). The SE difference images (top left corner) were adapted with permission from Shaheen *et al.*, J. Phys. Chem. Lett. **10**, 1960 (2019). Copyright 2019 American Chemical Society.^23^

To better describe the experimental set-up of LVS-UEM, it is necessary to briefly review the set-up of S-UEM with 30 kV as the applied AV. A schematic description of the second-generation S-UEM built in KAUST is provided in Fig. 2; detailed information has been previously reported.^34^ Briefly, it comprises two parts. The first is a fiber laser system with a fundamental wavelength of 1.03 *μ*m, a femtosecond amplifier with pulse width of 270 fs, and variable repetition rates ranging from 200 kHz to 25 MHz (Clark-MXR). A beam splitter separates the fundamental IR laser into two identical beams that pass through second and third harmonic generators (Clark-MXR) to generate a green beam (515 nm) for surface excitation and a UV beam (343 nm) for pulsed electron generation. The two harmonic beams are integrated into a modified SEM (Quanta 650, FEI). The green beam (pump) is directed onto the specimen in the SEM specimen chamber by a focal lens and produces an oval spot of 40 *μ*m in size. Similarly, the UV light is precisely guided and focused on the ZrO_2_-coated tungsten tip of a cold Schottky field-emission gun in the SEM to generate primary electron (PE) pulses (probe). Finally, a computer-controlled optical delay line (DL) is introduced to enable the time difference between the pump and probe pulses, with a time window of −0.60 to 6 ns. The PE probe scans the specimen's surface (both the illuminated and unilluminated regions) prior to or after the green pump beam at a designated time to release SEs from the uppermost surface of the specimen (a few nm depth), which are then captured for imaging by an Everhart–Thornley detector. Images at each timing are taken for 64 frames with a 300 ns dwell time; these are averaged as one image and used to deduct a single-frame image taken from a very negative time (such as −0.6 ns) to produce a “difference” image. The repetition rate is set at 8 MHz to enable a time interval of 125 ns at each sequential event and to ascertain the photoexcited region return to the original state before the next pulse arrives. A train of these difference images can visualize the charge carrier dynamics of photoactive materials.

As shown in Fig. 2, the main difference in experimental set-up between LVS-UEM in KAUST and the high-voltage S-UEM is that we introduced an extra optical delay line (a 1 keV kit; see the orange box in Fig. 2). This is based on the theoretical principle demonstrated in Eq. (1): at low AV, such as 1 kV, the PE pulses (probe) are accelerated at a slower velocity and arrive at the specimen's surface later than those at 30 kV,

$$v=\sqrt{\frac{2eV}{m_{0}}},$$ (1)

where *ν* (m/s) is the velocity of PE, *e* (in coulombs) and *m_0_* (kg) are the charge and invariant mass of PE, respectively, and *V* (in volts) is the applied AV of SEM. The relativistic effect was ignored here because the velocity of PE at low AV is much less than the speed of light (c) (ν_1kV_ = 1.87 × 10^7^ m/s, 6.25% of c) while, at 30 kV, the calculated (ca.) ν_30kV_ = 1.027 × 10^8^ m/s amounts to 34.2% of c, so the relativistic effect should be counted as being under 30 kV, and, according to Albert Einstein's special theory of relativity,^70^ the relativistic mass (m′ = m_0_ × [1 − ν^2^/c^2^]^−1/2^) of an electron should replace the invariant mass (*m_0_*) in Eq. (1), so Eq. (2) was deducted accordingly,

$$v=\sqrt{\frac{2eV}{m_{0}}{\left(1-\frac{v^{2}}{c^{2}}\right)}^{\frac{1}{2}}},$$ (2)

where *c* is the speed of light (c = 2.99 × 10^8^ m/s), the remaining physical parameters and their units are the same as in Eq. (1), and, at 30 kV, ca. ν_30kV_ = 9.977 × 10^7^ m/s.

In the S-UEM configuration, as mentioned before, the PE pulses that originate in the field emission gun are accelerated by an external electric field in the SEM apparatus and act as a probe to scan the sample surfaces; thus, the distance the PE travel at 30 kV and 1 kV is identical at a settled working distance of the SEM. However, based on the above calculations, the velocity of PE arriving at the surface of a specimen at 30 kV is 5.34 times that at 1 kV, which means that the average velocity at 1 kV is much less than that at 30 kV. Thus, in the case of 1 kV, to compensate for the delayed arrival of the PE, the pump laser beam must travel a longer distance to synchronize the arrival of the pump and probe (time zero of the S-UEM) at the surface of the specimen. To this end, two pairs of parallel mirrors (see the 1 keV kit in Fig. 2) are introduced as an extra delay line, and the mutual positions/distances among them are carefully adjusted by physically moving them back and forth until the time zero on the optical delay line (DL, as shown in the pathway of the green beam) reaches a position identical or very close to the one at the 30 kV configuration. It is worth noting that 1 kV is only an example presented here for the LVS-UEM; any other AV in the range of 1 to 30 kV can also easily be realized through a similar modification of the experimental set-up. For instance, Tagliaferri and colleagues recently developed an S-UEM apparatus with the AV at 5 kV to visualize the photoinduced evolution of surface potentials on vulnerable hybrid perovskite without causing permanent damage; the temporal resolution was limited to millisecond range.^46^

## APPLICATIONS OF LOW VOLTAGE EXPERIMENTS

**FIG. 3.**
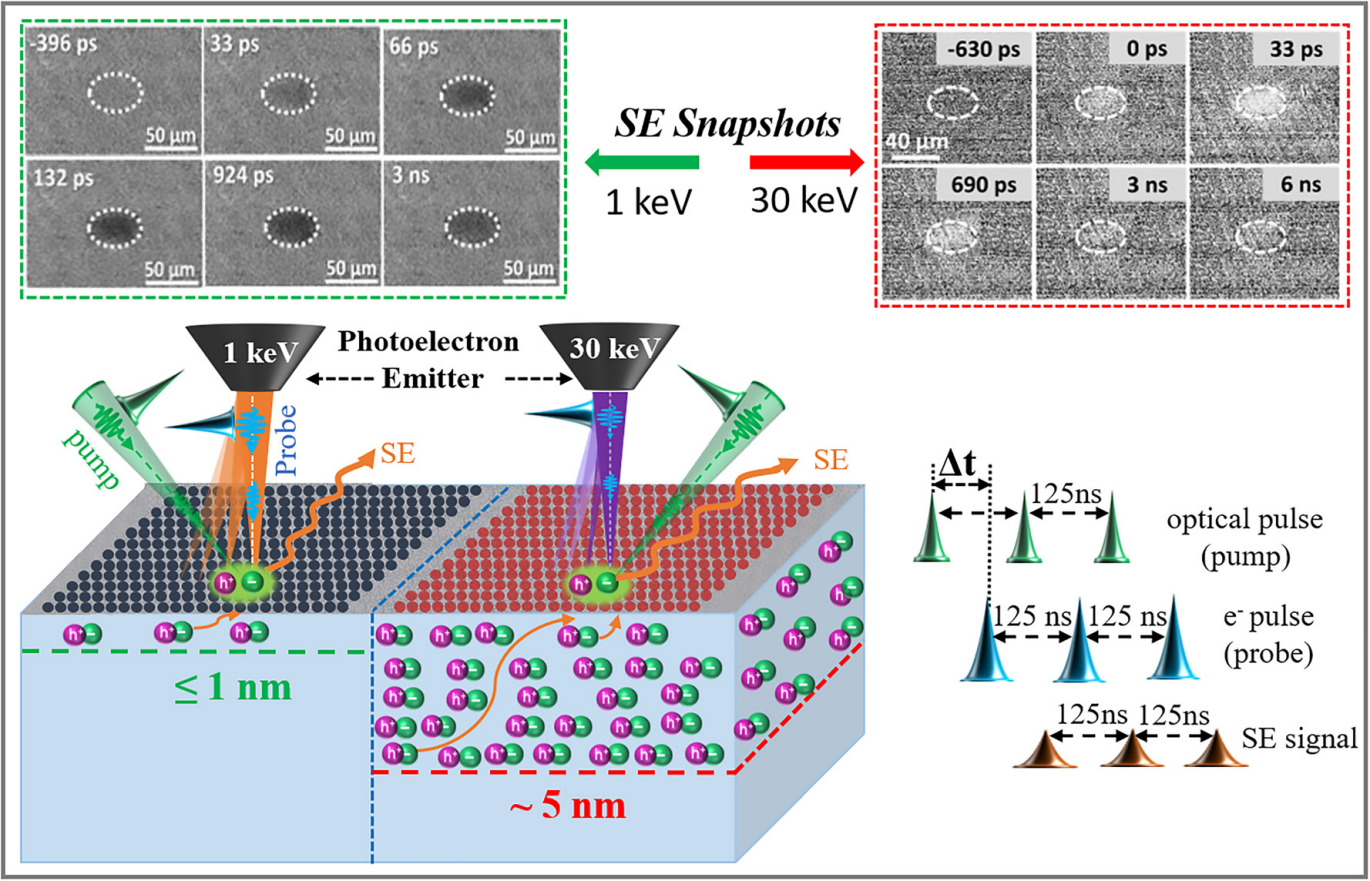
Comparison of the characteristics of S-UEM at high and low AV on an as-received Si (100) single crystal. The SE snapshots at 30 keV were adapted with permission from Shaheen *et al.*, J. Phys. Chem. Lett. **10**, 1960 (2019). Copyright 2019 American Chemical Society.^23^ The SE snapshots at 1 keV were adapted from Shaheen *et al.*, ACS Appl. Mater. Interfaces **6**, 7760 (2020). Copyright 2020 American Chemical Society.^72^

To confirm that our modified method can differentiate the uppermost surface dynamic information in real space and time, we used it to evaluate the influence of native oxide layers on the surface charge carrier dynamics of n-type Si (100) single crystals as a model system. As noted, there is an ultrathin (≤1 nm) SiO_2_ layer on the top surface of an as-received Si sample,^64^ and, even after complete removal of the oxides by HF etching, it regrows in minutes after exposure to air and moisture.^23,71^ As shown in Fig. 3, under identical experimental conditions (with the exception of changing the AV from 30 kV to 1 kV), the SE difference images at sequential time delays show a conversion from low contrast at 30 kV to high contrast at 1 kV in the laser illuminated area (the dashed ovals represent the footprint of the focused pump beam). Low contrast (energy gain) means that more SEs escaped from the photoexcited area and were detected compared to the unexcited one, while high contrast (energy loss) suggests that fewer SEs escaped and were detected, probably because they were trapped or scattered before they could emerge.^23^ This is verified by the experimental results that, after the removal of the surface oxide layers, the LVS-UEM at 1 kV produced low contrast in the photoexcited region.^72^ These results indicate that LVS-UEM at 1 kV can provide surface dynamic information in the angstrom range (≤1 nm) while S-UEM at 30 kV is less sensitive to the charge carrier behavior in the very top layers of materials' surfaces.

To obtain theoretical insights into the understanding of diverse surface dynamic information obtained at 30 kV and 1 kV, we calculated the information depth (*d*_id_) (or most probable escape depth) of Si (100) single crystals at 30 and 1 kV using Eqs. (3) and (4) (see the detailed description in Ref. 72); the calculated values were 4.54 nm and 0.91 nm, respectively. These values are in excellent accord with published theoretical^73–75^ and experimental results,^76,77^

$$d_{id(nm)}=0.267\frac{AI}{\rho Z^{2/3}}\;\left({5\;\mathrm{kV}\leq E}_{0}\leq 1\;\mathrm{MV}\right),$$ (3)

$$d_{id(nm)}=0.0534\frac{AI}{\rho Z^{2/3}}\;\left({0\;\mathrm{kV}<E}_{0}<5\;\mathrm{kV}\right),$$ (4)

where *A* represents the atomic mass (g/mol), *ρ* is the density (g/cm^3^) of the sample, *Z* represents the atomic number, *I* is the first ionization energy or electron affinity (eV) of the samples, and *E*_0_ is the AV (kV).

The experimental results and calculations show that it is reasonable to conclude that the interaction volume of S-UEM at 30 kV between the photoexcited region on the Si sample (at a depth of a few micrometers^21^) and the electron region (depends on the penetration depth of the PE probe beam) results in an information depth for SE of around 5 nm, which is much larger than the thickness of native oxides. This is probably why S-UEM at 30 kV is not sensitive enough to offer detailed information on charge carrier dynamics at the atomic or sub-nm surface level. By contrast, the energy of the PE generated by LVS-UEM at 1 kV is much lower (1 keV) and permits only SEs that originate at a depth of less than 1 nm under the material surface to escape. This region perfectly overlaps with the native oxide layers, and the small number of SEs carrying low energy (a few eV^77^) can be trapped effectively by the native oxide (SiO_2_) layer, resulting in a high contrast.

**FIG. 4.**
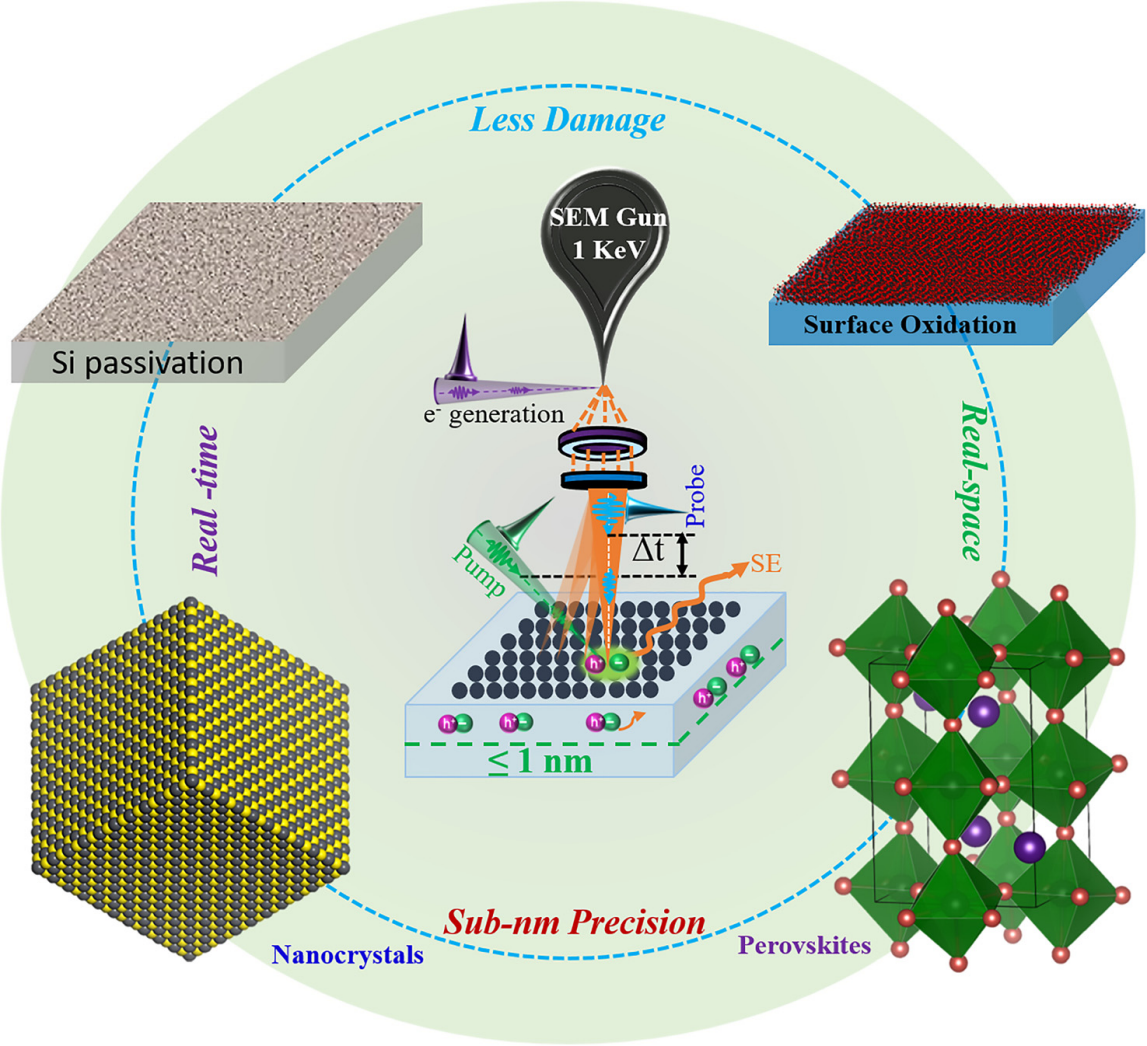
Potential applications of LVS-UEM as a surface-sensitive characterization technique.

To demonstrate its broad range of applications, we used the LVS-UEM to study the effects of surface native oxides in several important photovoltaic materials, such as CdZnTe, CdTe, and GaAs single crystals. The results obtained in all these materials showed high contrast in the presence of native oxides and changed into low contrast in the freshly cleaned, oxide-free surfaces of the same materials despite dissimilar orientations and/or doping conditions.^72^ Note that many other materials systems, such as two-dimensional materials, semiconductor nanocrystals, and perovskite, may also be studied by LVS-UEM as proposed in Fig. 4. This is because, under designated experimental parameters, such as a PE probe with low energy (as low as 1 keV), the permanent damage to the materials caused by electron irradiation turned out to be avoidable. These characteristics of LVS-UEM further enhance its ability to measure the surface properties of photoactive materials, which are generally very sensitive and vulnerable to electron irradiation. In other words, these results demonstrated the excellent sensitivity and selectivity of LVS-UEM to the ultrathin surface native oxides and further suggest that it is a promising method-of-choice for mapping the uppermost surface dynamics of photoactive materials. With the extremely shallow escape depth of SEs (≤1 nm) which can easily be further extended to several nm by rising AV, LVS-UEM will be versatile to explore the thickness-dependent surface dynamics of various 2D materials.^78^ It will also be a more powerful approach to track the dynamical evolution of surface acoustic wave on semiconducting polymers as compared to 30 keV experiment^47^ due to its superior surface selectivity. By using LVS-UEM, one can now visualize the deformation kinetics of hybrid perovskites without causing permanent damage that happened at 30 keV. Additionally, LVS-UEM may also have promising applications in exploring the photo-induced magnetization dynamics of ferroelectric materials.

## OUTLOOK

The progress made in 4D electron imaging over a decade of development and applications in both the scanning and transmission modes of operation is truly impressive. For instance, a fundamentally new understanding of ultrafast charge carrier dynamics on photoactive semiconductor surfaces and interfaces has recently been achieved through direct-surface imaging in real space and time through the use of a unique instrument: the high voltage four-dimensional scanning ultrafast electron microscope (4D-SUEM), which is the only instrument of its kind currently in operation. In this context, low voltage S-UEM provides an additional unique ability not only to map surface and interface dynamics at the sub-nm level but also to understand the impact of native oxide layers and any atomic-level surface treatment or passivation on their ultrafast charge carrier dynamics. Compared to conventional transient absorption spectroscopy or microscopy, ultrafast thermo-modulation microscopy, transient photocurrent measurements, and other apparatus based on pump-probe concepts, which either lack surface sensitivity or have limited resolution in time and space, LVS-UEM offers superior surface sensitivity, excellent spatiotemporal resolution, and a broad scope of application. Finally, the diverse applications discussed here make it clear that the low voltage methodology has the potential to characterize extreme surface properties of the photoactive materials commonly used in optoelectronic devices, such as perovskite and semiconductor nanocrystals, which have barely been studied using 4D imaging, especially after surface engineering. We conclude that most surface material properties can now be directly visualized in space and time by the LVS-UEM, including not only carrier dynamics, such as charge carrier transfer, separation, diffusion, and carrier trapping, but also other properties that are limited to the surface, such as photoinduced surface potentials and surface mechanical properties.
